# Rheumatoid arthritis in Latin America. Important challenges to be solved

**DOI:** 10.1007/s10067-015-3048-1

**Published:** 2015-08-09

**Authors:** Ruben Burgos Vargas, Mario H. Cardiel

**Affiliations:** Department of Rheumatology, Hospital General de México, Faculty of Medicine, Universidad Nacional Autónoma de México, México City, Mexico; Centro de Investigación Clínica de Morelia, Virrey de Mendoza 1998. 522. Col. Félix Ireta, Morelia, Mich Mexico 58070

Since the introduction of biologic agents for the treatment of patients with rheumatoid arthritis (RA), there have been enormous advances in clinical and therapeutic aspects of the disease. Likewise, there is now much more information on the role of genetics and environmental factors in the pathogenesis of the disease. Throughout the years, the main therapeutic target is the control of disease activity as soon as possible to prevent structural damage and functional consequences.

RA is a diagnosis that is not easily recognized at early phases. Lay people associate rheumatic diseases with old age, minor physical trauma, weather, dietetic conditions, or emotional issues. It is not a surprise that many patients seek medical care at late stages, when irreversible damage has already occurred.

Sustained remission or low-disease activity levels without joint space loss and erosions have become the main objective of current therapies. Health-related quality of life (HRQoL) improves and long-term socioeconomic consequences decreases.

Primary care physicians are usually the first medical contact. These clinicians should have special tools for early diagnosis, proper referral, and initial treatment in all patients with a clinical suspicion of RA. This is not the case in many countries where rheumatology training in undergraduate programs is not fully implemented.

Rheumatology societies in Latin America should have a more active participation in medical education in undergraduate programs including practical activities. This will be probably an important investment to diminish the burden of illness that RA causes to the society [1].

Another challenge that many countries face is having an insufficient number of rheumatologists. As expected, rheumatologists usually have busy clinical activities. The time devoted to individual care is not enough to establish an adequate relationship, consultations are not usually as frequent as they should be, medical resources are not always what medical guidelines suggest, and patients tend to receive a suboptimal medical care. This should motivate rheumatology organizations to identify intelligent and practical alternatives to improve medical care despite a shortage of rheumatologists. Education, innovation, and coordination seem to be the key activities to be done.

Once a clinical diagnosis has been made, clinicians should be aware that low educational level in RA patients in Latin America has been associated with higher disease activity, more radiologic progression, and poor clinical outcomes. This is probably related to late diagnosis and some practical and therapeutic variables [2]. All patients and particularly those with a low educational level require special health education programs.

Patient education in rheumatoid arthritis is an urgent need to discuss what is expected of medical and nonmedical treatments and the importance of patient and family participation and improve patient-doctor communication skills.

Adherence to medical treatment in chronic diseases remains a very important problem in the world. It is recognized that poor adherence is associated with worse clinical and radiological outcomes [3].

Costs of medication impose an important economic burden to patients and their families [4]. Comorbidity in rheumatoid arthritis increases complexity to medical care. In many cases, rheumatologists are the team coordinators and should be prepared to offer proper management. There is a need to implement therapeutic strategies that include a more comprehensive medical care.

It is now 20 years since the first report on the efficacy of a monoclonal antibody—infliximab—to tumor necrosis factor alpha (TNF-α) in patients with RA [5]. Compared to placebo, the effect of the TNF-α blocker was impressive and authors concluded that such “results provide the first good evidence that specific cytokine blockade can be effective in human inflammatory disease and define a new direction for the treatment of rheumatoid arthritis.”

That new direction has benefited thousands of patients all over the world through the implementation of programs for early recognition of the disease, redefinition of classification criteria, improved designs of protocols for the study of the efficacy and safety of biologics and DMARDs, continuous increase in the number of agents—many directed to new targets, the instauration of biologic registries, and post-marketing surveillance.

It is clear that the implementation and maintenance of such programs require a considerable budget to cover the cost of the infrastructure needed to accomplish their goals and the cost of biologics itself. While most European countries relay in the percentage of their gross domestic product (GDP) devoted to health care to comply with those programs, in other parts of the world, including Latin America, the cost is partially covered by a variety of entities, including patients’ and households’ own pocket money. Similar or even worst situations occur in Africa and Asia resulting in a tremendous gap between developed and underdeveloped countries regarding the identification of RA and its current treatment. In this sense, the implementation of programs for early detection and treatment of RA might be less successful than in developed countries.

Health care in Latin America is typically fragmentary [6]. In some countries, the state provides health care for the whole population while in others, individuals pay for their medical expenses. Reimbursement is very limited and is only available in some countries. Access programs are available in some countries where the government usually provides the drug. In this sense, it is not convenient to make any generalization of the characteristics of health care in Latin America. Barriers to implement early recognition of RA and treat them early on the course of the disease related to the healthcare system in Latin America include several aspects.

The nation’s GDP budget for health care had a sustainable increase in several Latin American nations. The mean percentage of Argentina, Brazil, Chile, Colombia, Mexico, and Venezuela increased from 7.9 % in 2009 to an estimated 8.1 % in 2017 [7]. The way each country handles their annual budget is unknown. In general, most of the budget goes to pediatric diseases, chronic degenerative conditions, and metabolic diseases reducing the individual life expectancy.

The situation here is that the percentage of such budget devoted to rheumatic diseases is probably very small and insufficient to run any health program at the national level. As a group, rheumatic diseases are not considered of clinical, socioeconomic, and cost relevance for the state. It is therefore neither as a group nor individually deserve the attention of health authorities whom apparently consider that rheumatic diseases are not a health problem.

The role of the state—represented by healthcare system—in implementing early arthritis clinics is limited. The cost of such a program includes some infrastructure, human resources, and effective treatment. The prevalence of RA in Latin America ranges between 0.4 and 1.6 % [8–10].

At least in the Mexican population, there are variations in the prevalence of RA related to geographic regions that might be explained by the low educational and socioeconomic level represented by people who only speak a native language.

In contrast to what health authorities consider, epidemiological data supports the idea that RA is not a rare disease and that late diagnosis is associated to bad prognosis.

It is possible that at the institutional level, the facilities to carry on an additional clinic, specifically an early arthritis clinic and human resources, would be impossible. Most clinics, physicians, and nurses are overwhelmed by daily consultations. Despite the fact that needs would depend on the success of the program, some challenging situations must be solved.

The infrastructure requires the space needed to take care of patients with early disease. Space limitations may account for inadequate care and communication between the patient and the physician. There should be access to laboratory examinations, imaging studies, infusion rooms, and refrigeration systems.

Human resources include physicians—from general practitioners to rheumatologists, nurses, joint counters, and some administrative people. They should be paid for their services and should be continuously trained.

The design of protocols according to the procedures to be performed should be clear and well justified.

Similarly, the clinic should have the capacity to provide the patient with the medication he needs. Despite its popularity, recommendations for treating patients with RA according to international standards, including the American College of Rheumatology/European League Against Rheumatism recommendations [11] and the Treat to Target initiative [12], its implementation in clinical practice might be difficult in the presence of some of the barriers mentioned before. Early recognition and proper assessment of the clinical status of the patient does not mean that medications would be available for everyone.

In summary, recognition and therapeutic advances in RA have evolved very rapidly in the last 20 years. Unfortunately, alongside with these advances, a gap between fragmented and socialized healthcare services is becoming wide. And that seems to be the case for some Latin American countries.

We have identified the most important challenges that patients, doctors, and medical systems face to improve the medical care of one of the most important rheumatic diseases. RA will remain a health problem despite medical advances if we do not effectively act on these challenges to modify the outcome in most RA patients in Latin America.
